# Characterizing the Performance of a Compact BTEX GC-PID for Near-Real Time Analysis and Field Deployment

**DOI:** 10.3390/s21062095

**Published:** 2021-03-17

**Authors:** Isis Frausto-Vicencio, Alondra Moreno, Hugh Goldsmith, Ying-Kuang Hsu, Francesca M. Hopkins

**Affiliations:** 1 Department of Environmental Sciences, University of California, Riverside, CA 92521, USA; amore022@ucr.edu (A.M.); fhopkins@ucr.edu (F.M.H.); 2 SRI Instruments, Torrance, CA 90503, USA; hugh@srigc.com; 3 California Air Resource Board, Sacramento, CA 95814, USA; ying.hsu@arb.ca.gov

**Keywords:** BTEX, benzene, air toxics, gas chromatography, traffic emissions

## Abstract

In this study, we test the performance of a compact gas chromatograph with photoionization detector (GC-PID) and optimize the configuration to detect ambient (sub-ppb) levels of benzene, toluene, ethylbenzene, and xylene isomers (BTEX). The GC-PID system was designed to serve as a relatively inexpensive (~10 k USD) and field-deployable air toxic screening tool alternative to conventional benchtop GCs. The instrument uses ambient air as a carrier gas and consists of a Tenax-GR sorbent-based preconcentrator, a gas sample valve, two capillary columns, and a photoionization detector (PID) with a small footprint and low power requirement. The performance of the GC-PID has been evaluated in terms of system linearity and sensitivity in field conditions. The BTEX-GC system demonstrated the capacity to detect BTEX at levels as high as 500 ppb with a linear calibration range of 0–100 ppb. A detection limit lower than 1 ppb was found for all BTEX compounds with a sampling volume of 1 L. No significant drift in the instrument was observed. A time-varying calibration technique was established that requires minimal equipment for field operations and optimizes the sampling procedure for field measurements. With an analysis time of less than 15 min, the compact GC-PID is ideal for field deployment of background and polluted atmospheres for near-real time measurements of BTEX. The results highlight the application of the compact and easily deployable GC-PID for community monitoring and screening of air toxics.

## 1. Introduction

The volatile organic compound (VOC) family of benzene, toluene, ethylbenzene, and xylene isomers (BTEX) are air pollutants that can cause detrimental health effects and degrade air quality through oxidation reactions [1,2]. BTEX compounds are monocyclic aromatics and are grouped together because of similarities in their structures, properties, and emission sources [3]. These compounds are emitted as combustion byproducts of fossil fuels and biomass, including motor vehicles and wildfires, and through volatilization from crude oil or its derivatives, including gasoline and industrial solvents [4,5,6,7]. BTEX compounds are classified as hazardous air pollutants (HAPs) and are regulated by a large number of agencies worldwide including the United States Environmental Protection Agency (US EPA) and the California Air Resources Board (CARB) [8,9].

Among the BTEX family, benzene is the most dangerous as it is a well-known carcinogen and may have adverse health effects on immune, metabolic, respiratory functioning as well as on development [10,11,12,13]. Ethylbenzene has been classified as a possible carcinogen, while toluene and xylene isomers can cause damage to the brain and central nervous system with long term exposure [10,14,15,16]. BTEX is ubiquitous in the environment at trace levels ranging from sub-ppb to tens of ppb in urban and industrial areas where atmospheric mixing ratios are higher [4,17,18,19,20,21,22]. Although ambient atmospheric BTEX levels have dropped due to reformulation of gasoline [23], there is evidence of an increase of emissions from oil and natural gas operations [24,25]. As wildfire events become more common with climate change, exposure to BTEX may increase in rural areas [26,27].

Monitoring of BTEX atmospheric background levels requires instrumentation that is sensitive to sub-ppb levels [17,19]. Current techniques for measuring BTEX include ultraviolet (UV) spectroscopy, infrared (IR) spectroscopy, and gas chromatography (GC) coupled either to a flame ionization detector (FID), photoionization detector (PID) or to a mass spectrometer (MS) [28,29,30,31]. Traditional methods require ambient air samples to be drawn into sorbent material or collected in evacuated stainless-steel canisters then transferred to the lab for further analysis. More recently, open path Fourier-transform infrared spectroscopy (FTIR) and proton transfer reaction-mass spectroscopy (PTR-MS) have allowed for near-real time analysis in the field [32]; however, these techniques are expensive to purchase and operate due to their need for support gases, power requirements, or large physical size, and hence are not ideal for long-term stationary or mobile monitoring [30,33,34,35,36].

There is currently a need for more inexpensive, easy-to-operate screening methods to determine the presence of atmospheric BTEX levels, as elucidating the fine-scale spatial patterns of BTEX in populated areas can improve the accuracy of human exposure estimates for the surrounding communities and inform mitigation policy [9]. The California Assembly Bill 617 calls for community-focused monitoring in disadvantaged and highly impacted communities [37]. This bill and the existing technology have popularized the use of inexpensive sensors to provide an accessible screening method for communities due to the accessible prices and portability (e.g., Purple Air, Clarity, etc., for particulate matter pollution) [37,38]. Numerous inexpensive sensors for BTEX and VOCs have been designed [38,39,40,41]; however, very few have the combination of sub-ppb sensitivity, selectivity, and relative low cost needed for ambient air monitoring. See Spinelle et al., 2017 and Lara-Ibeas et al., 2019 for a summary of the latest laboratory prototypes and commercially available inexpensive BTEX sensors and GC’s [38,39].

As a common inexpensive option, PID can be used as a standalone instrument to measure total hydrocarbon presence in real time. Although PIDs have great sensitivity, they are not selective and cannot speciate VOCs. Pairing a PID with a GC allows for speciation of BTEX compounds and lower detection limits. In this study, the performance of an ultra-compact GC-PID is characterized for detecting BTEX at sub-ppb levels. The instrument configuration is optimized for separation of BTEX compounds. This analytical instrument was developed for operation in the field to be used as a screening tool for onsite and near-real time analysis. This design uses ambient air as the carrier gas to minimize the need for support gases and a calibration strategy is established that is simple and requires minimal equipment. The compact BTEX GC-PID system is composed of the following modules: sampling, preconcentration, separation, and detection described in the following Section 2.1.1, Section 2.1.2, Section 2.1.3, Section 2.1.4 and Section 2.1.5. The instrument was characterized in a laboratory setting (3.1) and was tested in the field (3.2). Section 4 discusses findings and offers recommendations followed by concluding remarks in Section 5.

## 2. Materials and Methods

### 2.1. Prototype of a Compact BTEX GC-PID System

**Figure 1:**
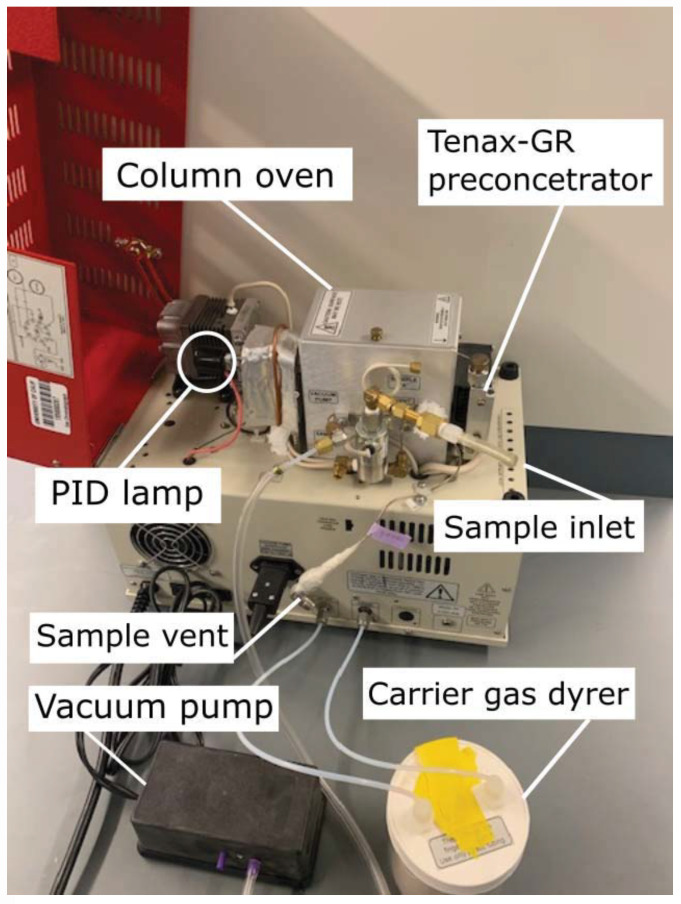
Side view of the compact benzene, toluene, ethylbenzene, and xylene isomers (BTEX) compact gas chromatograph with photoionization detector (GC-PID) prototype. The system has an ultra-compact chassis (SRI 110 chassis model) weighing 15 kg with dimensions of 36.8 × 21.6 × 34.3 cm.

The compact BTEX GC-PID system is composed of the following modules: sampling, preconcentration, separation, and detection, further described in the following Section 2.1.1, Section 2.1.2, Section 2.1.3, Section 2.1.4 and Section 2.1.5. The GC system was developed by SRI Instruments (Torrance, CA, USA) and is based on a simpler version of the commercial SRI BTEX GC-PID-FID with a built-in Method 5030 compliant purge and trap concentrator. The modified BTEX GC-PID design (Figure 1) has the advantage of a reduced size and weight (SRI 110 chassis model) that allows it to be field-deployable and convenient for measurements. The instrument weighs 15 kg with dimensions 36.8 × 21.6 × 34.3 cm. It is designed for field deployment in background and polluted atmospheres with automatic sampling every 12–15 min. The GC-PID instrument operates in isothermal mode where the BTEX molecules separate without an oven temperature ramp. BTEX concentrations measured by the instrument are reported as mixing ratios defined as the ratio of the mass of the respective BTEX compounds to the mass of dried air in a given volume.

**Figure 2:**
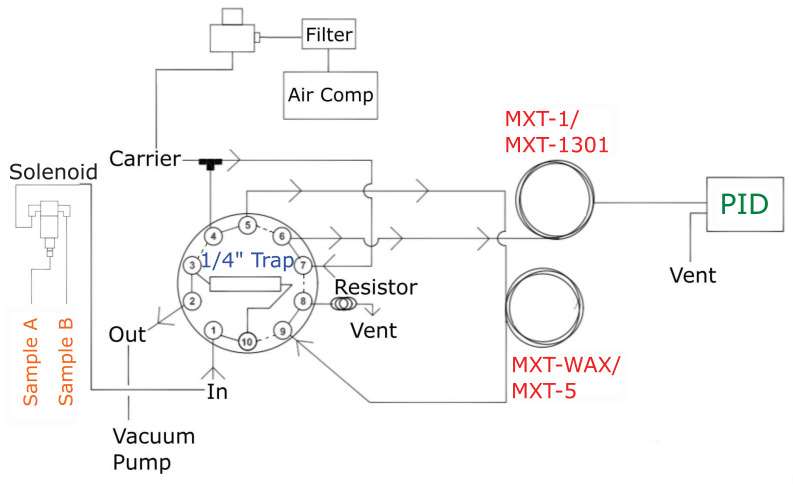
Schematic of the compact GC-PID system operating principle with 10-port valve in “load” position (shown as the solid lines) and “inject” position (shown in the dotted line) in a precolumn backflush configuration. Principal components of the different modules are shown in different colors: sampling (orange), preconcentration (blue), separation (red) and detection (green).

A prototype GC was built by SRI Instruments and tested. Initially, the prototype was built with a distinct configuration to test for the desired sub-ppb limit of detection, selectivity and separation for monitoring BTEX in ambient air. The column and backflush configurations tested is further discussed in Section 2.1.4. Ultimately, the configured system operates as follows: preconcentration of sample matrix on Tenax-GR material, separation by 15 m MXT-5 and 30 m MXT-1301 columns (Restek), followed by PID detection at 10.6 eV. Instrument parameters and settings are modified with the PeakSimple software downloadable on the SRI Instruments’ website. The instrument operating principles are represented in the schematic shown in Figure 2. Two instruments were tested with this configuration, which we henceforth refer to as GC1 and GC2.

#### 2.1.1. System Integration and Instrument Operation

The instrument operating principles are represented in the schematic shown in Figure 2 in a precolumn backflush configuration. When the 10-port valve is in “load” position, the vacuum pump pulls ambient air in through the solenoid valve set to sample from either inlet A or B. This is then directed to the Tenax-GR trap to load the BTEX sample at the adsorption temperature (40 °C). Any sample not adsorbed to the Tenax-GR material is vented through the “Out” port. The trap is heated to the desorption temperature (180 °C) shortly thereafter. At the same time, carrier gas has been flowing through the columns at a constant flow rate defined by the electronic pressure controller. Once the trap reaches the desorption temperature (180 °C), the valve is actuated to “inject” position where the carrier gas is directed towards the trap sweeping desorbed analytes into the analytical columns (labeled “MXT-WAX/MXT-5” and “MXT-1/MXT-1301” in the diagram). The desorbed analytes are separated by boiling point before reaching the photoionization detector (labeled “PID”). The PeakSimple software displays a chromatogram in real time with automatic detection of peaks, integration and concentration calculation using a component and calibration file that has been saved prior to sampling. The BTEX molecules appear on the chromatograms based on their boiling point temperatures with benzene first followed by toluene, ethylbenzene, co-eluted meta- and para-xylenes (m,p-xylene) and finally ortho-xylene (o-xylene). The PeakSimple software allows the operator to modify instrument parameters and settings for desired sampling time, modification of event tables, calibration and manual integration of peaks.

#### 2.1.2. Sampling Module

The sampling module consists of an aquarium vacuum pump that pulls the sample matrix into the Tenax-GR trap (Figure 1). The two brass inlets (1/8” (3.175 mm) female) are connected to a two-position solenoid valve that allows for alternating measurements between a calibration standard and an atmospheric sample. The two-inlet option can be used for faster sampling as the solenoid valve switches between loading a sample and finishing the previous loaded sample or a calibration gas standard. A plastic male barbed hose fitting with 2.5 cm long Teflon tube was connected to the brass inlets for all experiments and calibrations conducted in this study.

#### 2.1.3. Preconcentration Module: Tenax-GR Trap

The preconcentration module consists of a ¼” × 4 ½” (6.35 × 114.3 mm) stainless-steel cylinder packed with 0.5 g Tenax-GR material (2,6-diphenylene-oxide polymer resin). A volume of gas is pulled through the solenoid valve by a vacuum pump into the Tenax-GR trap. This concentrates the desired volume of sample, trapping volatile organics while largely excluding water before loading the gas into the column. The amount of sample that may be loaded on to the trap is limited by the trap’s adsorbent packing. The packing of the trap with the Tenax-GR sorbent material may affect the flow rate, thus flow rates for each instrument (GC1 and GC2) were determined to ensure the same sampling volume of 1 L. Sampling times of 1.75 and 2.9 min were established for sampling 1 L volume for GC1 and for GC2, respectively.

During trapping, ambient air or standard gases are flowed through the trap at 40 °C, until 1 L of this sample matrix has been passed through, depending on the flow rate of each instrument. The trap is then heated to an optimal temperature of 180 °C maintained for 4 min to allow thermal desorption of BTEX molecules from the Tenax-GR material. The heating system consists of a thermocouple wire and aluminum block with a 100-watt cartridge heater wrapped around the Tenax-GR trap stainless steel tubing with a temperature ramp of 180 °C/min. The trap is then cooled by a small fan within 3.45 min to 40 °C.

#### 2.1.4. Separation Module

The separation module consists of two coupled columns heated in a small air-bath oven at 60 °C. The oven houses the two columns, a small fan, and a 10-port gas sampling valve (Figure 1) that connects the entire system further described in schematic shown in Figure 2. The small fan circulates air inside the oven to keep an equal distribution of heat. A syringe injection port is included on the side of the oven wall to bypass the Tenax-GR trap, in cases when direct gas sampling is preferred.

Various column configurations and flushing methods were tested to optimize separation of BTEX with a stable baseline while maintaining a low cost for the GC measurement system. Table 1 describes the columns and flushing methods tested, labeled below as configuration a, b, and c.

- Precolumn backflush to vent (configuration a and c): This method captures heavier molecules in the precolumn and prevents them from entering the analytical column and reaching the detector. The backflush is carried out at a user defined time to reject water and other high boiling point analytes while the analytical column runs at a constant flow. This configuration has the advantage of the sample matrix having little influence on measurement, allows faster sampling time, prevents late eluting compounds from interfering with the subsequent runs, and prevents water in the sample matrix from reaching the column.
- Backflush to detector (configuration b): This method bundles C6+ components that elute to the detector after the molecules of interest have passed through the analytical column. This method reduces analysis time and presents a summed total of C6+ molecules displayed in the chromatogram. It also prevents late eluting compounds from interfering with the subsequent runs.

**Figure 3:**
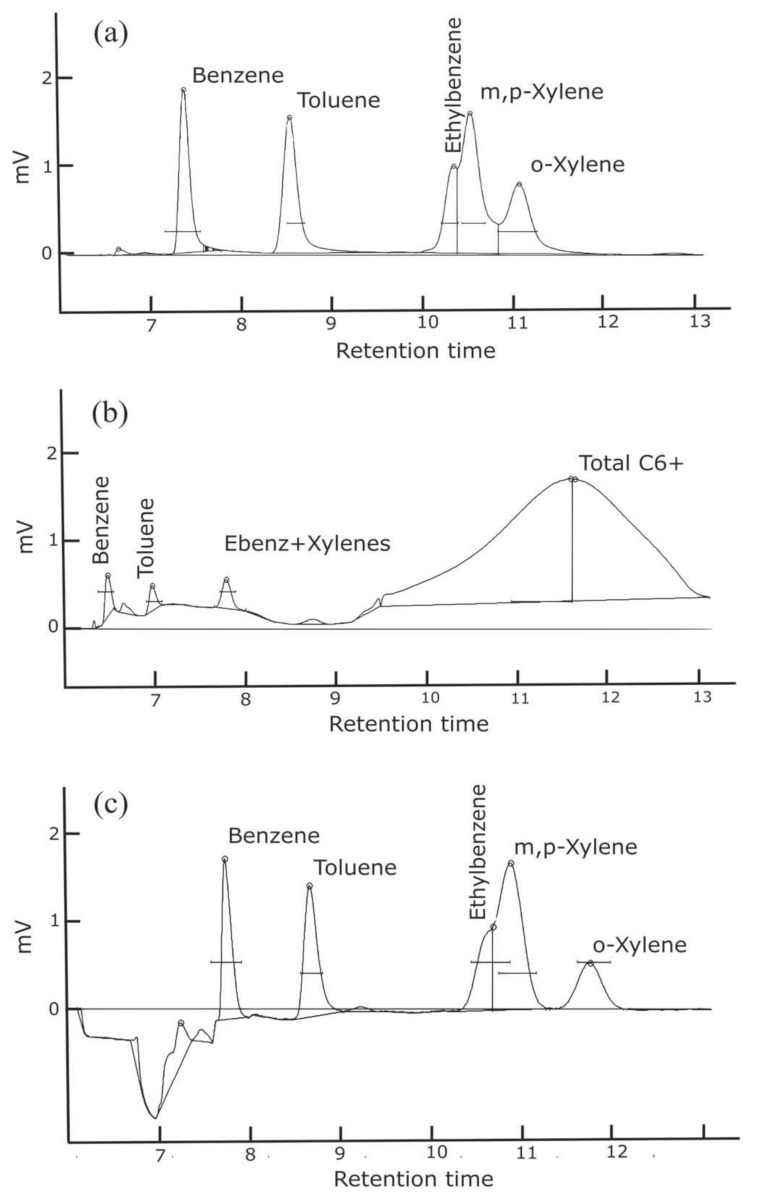
Sample chromatograms for three different column and flushing configurations with injection of a 1 ppb BTEX standard. Configurations: (**a**) Separation of heavier BTEX compounds is challenging with the selected columns. (**b**) Heavier hydrocarbons are detected; however, the baseline is not stable and separation of heavier BTEX compounds remains challenging. (**c**) There is better separation of the o-xylene; however, separation of ethylbenzene and m,p-xylenes still remains challenging. The baseline shifts when the 10-port solenoid valve rotates, and the precolumn configuration is no longer in series with the analytical column. This causes the column flow to increase and the baseline to shift as seen in (**c**).

Configuration a and b operated with the precolumn, a 15 m polar phase (0.53 mm ID × 2.0 µm MXT-WAX) capillary column and a 15 m long nonpolar phase capillary (0.53 mm ID, 5.0 µm MXT-1) analytical column. The MXT-WAX column helps to remove water and VOC’s other than BTEX. In this configuration, complete separation of the heavier BTEX compounds is challenging with the selected columns. This first configuration was optimized for separation of benzene; however, it did not entirely separate ethylbenzene and xylene isomers without needing an oven temperature ramp, as seen in Figure 3a. Configuration b had the same columns as configuration a, but was plumbed to backflush to detector. While the backflush to detector provided information on the number of hydrocarbons present in the sample, the baseline was not stable or consistent (Figure 3b). This leads to uncertainties when integrating the area of each analyte peak. Thus, we retained the precolumn to detector plumbing. Configuration c was plumbed with capillary columns MXT-5 with 15 m length (0.53 mm ID × 0.25 µm) and MXT-1301 with 30 m length (0.53 mm ID × 0.3 µm). The baseline proves to remain stable with a better separation of the o-xylene, however separation of ethylbenzene and m,p-xylenes remains challenging (Figure 3c). An oven temperature ramp is necessary to separate those two molecules; however, due to cost consideration the GC remained in isothermal mode with option c as the final configuration. Both GC1 and GC2 were sent back to the manufacturer to be configured with a precolumn backflush and capillary columns MXT-5 and MXT-1301.

#### 2.1.5. Detection Module

Once molecules are separated within the analytical column, the carrier gas directs the analytes toward the detector. The BTEX GC-PID instruments are equipped with a PID detector (Andrews Glass) that responds to compounds whose ionization potential is below 10.6 eV, this includes aromatics and molecules with double carbons. This particular PID has a krypton discharge lamp that fragments the VOC’s into negative and positive ions, the amplifier then measures the negative ions created by the lamp’s UV energy at 121 nm.

### 2.2. Gas Standards and Carrier Gas

A certified gas mixture composed of 1 ppm of each of the BTEX compounds (1 ppm of benzene, 1 ppm of toluene, 1 ppm of ethylbenzene, 1 ppm of m,p-xylene and 1 ppm of o-xylene) purchased from Restek (±5% uncertainty) and MESA Specialty Gases (±10% uncertainty) were used for experiments performed in the laboratory. In addition, we used a gas mixture of about 1 ppb of each of the BTEX compounds from Apel-Reimer Environmental, Inc. (±20% uncertainty), NIST-traceable certified, for the time-varying calibration method.

An internal air compressor provides the carrier gas from ambient air without the need for support gases (e.g., He, N_2_). The “Whisper Quiet” air compressor is built in the chassis of the GC and controlled by an electronic pressure controller maintained at 9 PSI (62 kPa). The stream of air passes through the Sample Stream Dryer (SRI P/N 8690-0152) housing a Nafion permeable membrane dryer (Permapure P/N ME 110-24-COMP4) contained in the blue indicating molecular sieve to remove water vapor and other impurities from the ambient air carrier gas. The Nafion tube was cleaned as needed following the manufacturer’s suggested procedure. The molecular sieve was heated regularly to the manufacturer’s recommended temperature to remove moisture from the desiccant beads as they turned brown when saturated.

An experiment was conducted to determine the percent loss of humidity by passing a moist stream of air (62.6% relative humidity) through the Sample Stream Dryer. The Nafion dryer in molecular sieve significantly reduced the humidity to 32.3% in the air stream by close to 50% percent decrease. See Section 3.1.5 for the effect of humidity on BTEX detection.

### 2.3. Calibration Methods

Two calibration methods were explored with the GC system using the PeakSimple calibration tool. The first involved diluting 1 ppm BTEX standard with gas tight syringes and/or mass flow controllers in zero air within Tedlar bags. This method required 1000-fold dilution of the 1 ppm BTEX gas standard in addition to delicate analytical tools which are not ideal to use in the field, thus the second method was preferred and used to characterize the instrument.

The second method explored was the time-varying calibration method that takes advantage of the flexibility in the trap loading time to control the amount of sample reaching the column and detector. This method relies on the fact that the trapping time on the Tenax-GR preconcentrator is linearly proportional to amount of sample loaded, and the area detected by the GC. The linearity of the calibration curve was explored to evaluate appropriate sampling volumes (and times) to cover the concentration range of interest (Section 3.1.2). This time-varying method involves less equipment and is ideal for long-term field campaigns where a standard gas can be programmed for automated measurements.

### 2.4. Field Deployments

We deployed the GC in a mobile platform by powering it with deep cycle marine batteries coupled to a pure sine inverter. Ambient air samples were drawn in from outside of the moving vehicle through Teflon tubing connected to the GC inlet. The GC draws a maximum of 180 W when the Tenax-GR trap is heated and 60–70 W when idle. For field measurements without the need of wall power, the system can be powered for more than 6 h by deep cycle marine batteries connected in parallel to a pure sine wave inverter. The low power consumption makes it an ideal instrument for mobile measurements where power is limited.

For outdoor deployments and humidity experiments, we used an OMEGA sensor (OM-CP-PRHTEMP101A) to record pressure, temperature, and relative humidity. A GPS tracker was used for mobile measurements to match sampling times with location.

## 3. Results

### 3.1. Instrument Characterization

Several experiments were conducted to characterize and optimize the performance of the BTEX GC-PID instruments for the detection of ambient levels of BTEX expected to be ~1 ppb. We evaluated the performance by studying the following instrument parameters: linearity of the system, detector signal vs. sample volume, limit of detection, instrument drift, and humidity effects in the sections below.

#### 3.1.1. Linearity of the System

**Figure 4:**
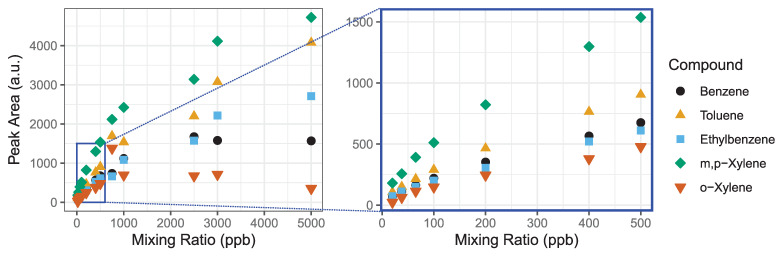
We evaluate the detection range of the BTEX GC-PID system by measuring a wide range of mixing ratios until an asymptote is reached. The detection linear range (0–500 ppb) is magnified highlighted by the blue box. The R^2^ values for linear fits to each compound are all greater than 0.98.

The detection range of the GC-PID system is limited by the adsorption capacity of the Tenax-GR trap in conjunction with the linear detection range of the PID lamp. The GC-PID system relies on the adsorption of BTEX molecules onto the Tenax-GR trap to preconcentrate the analytes for detection at trace levels. PID lamps have excellent sensitivity, detection limits and extensive linear detection ranges, but the latter begins to deviate at higher ppm levels. We test the linearity of the system as a whole with influences from both the Tenax-GR trap and PID detector to determine the maximum range of mixing ratios that is measurable with the instrument. We made a saturation curve by loading 1 L samples of BTEX with mixing ratios ranging from 20 to 5000 ppb (Figure 4). We investigate the detection limit further described in Section 3.1.3.

Using a mass flow controller, a 1 ppm BTEX gas standard in zero air was diluted into Tedlar bags, and then were analyzed on the GC-PID to make the saturation curve. Prior to each measurement a trap blank was performed to ensure analytes were removed prior to the next sample. The following concentrations were tested: 20, 38, 65, 100, 200, 400, 500, 750, 1000, 2500, 3000 and 5000 ppb (Figure 4). We observed that BTEX peak areas are linear at low mixing ratios, but curve toward an asymptote at higher mixing ratios as seen for all analytes by 1000 ppb. This experiment demonstrates that for a sampling volume of 1 L, measurements up to 500 ppb can be made with confidence. Higher mixing ratios will be underestimated due to combined effect of detector linearity limitation and saturation of the Tenax-GR trap. We explore further the linearity of these curves in the next Section 3.1.2 to estimate a range of measurement accuracy.

#### 3.1.2. Detector Signal vs. Sample Volume

We then evaluated the influence of varying sample volumes passing through the system to determine the range for the calibration curve using a time-varying method. At background, BTEX is expected to be below 1 ppb, while in a polluted atmosphere, mixing ratios vary greatly and can reach tens to hundreds of ppb. These experiments were focused on expected ambient BTEX concentrations of 1 ppb. The gas standard composed of 1 ppb of each of the BTEX compounds was loaded onto the Tenax-GR trap while varying the sample volume from 1 to 10 L representing a concentration range of 0.5 ppb to 5 ppb BTEX on the trap. A cleaning step was performed after each analysis to remove any BTEX molecules from the Tenax-GR preconcentrator and prevent influence on the following sample. The cleaning step involved heating the Tenax-GR trap for 3 min at the end of the analysis to vent out desorbed analytes followed by cooling of the trap. Blank runs verified that BTEX was fully desorbed before the subsequent analysis. Conducting a cleaning step proved to be an effective method for removing the effects of carry-over from near-background and polluted samples of over 100 ppb. One cleaning step removed 87.1% of carry-over, two cleaning steps removed 92.9% and four cleaning steps removed 95.6% of carry-over. Due to the effectiveness of one cleaning step, we apply this to experiments presented in this study. Cleaning steps were conducted as trap blanks and chromatograms were visually verified for significant removal of carry-over.

**Figure 5:**
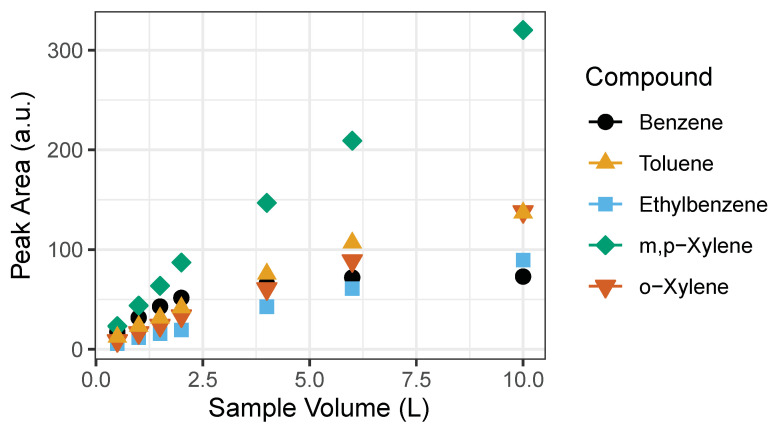
Linearity of detected signal to sampling volumes of BTEX compounds. Signal becomes saturated past 2 L, particularly evident for benzene. The error bars represent the standard deviation of the mean of triplicates of peak area. Note: error bars do not appear because they are smaller than the size of the symbol.

We found that past 2 L of sample volume the relationship between the benzene sample volume and instrument response ceases to be linear (Figure 5). Both GC1 and GC2 showed the same behavior. Probable cause may be that the Tenax-GR trap gets saturated towards a large sample volume. However, linearity in benzene exists at the lower end of the sample volume (0.5, 1, 1.5 and 2 L) as seen in Figure 5. For calibration purposes, we used volumes ranging from 0.5 to 2 L with 1 ppb BTEX gas standard corresponding to 0.5, 1, 1.5, and 2 ppb that represent expected ambient BTEX mixing ratios. A 1 L sampling volume was determined to be adequate.

**Figure 6:**
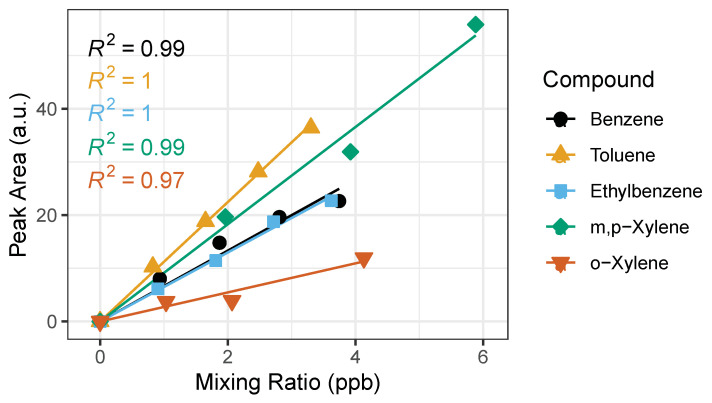
Example of calibration curves for BTEX compounds with the time-varying calibration method using sampling volumes: 0.5, 1.0, 1.5 and 2.0 L. The horizontal error bars represent uncertainty from the certified gas standard, while the vertical error bars (smaller than symbol) represent random instrument error. The corresponding R^2^ is shown for each BTEX compound.

Once an adequate range was established for the calibration curve with the time-varying calibration method at the range where linearity exists (0.5–2 L), a straight-line calibration model is evaluated. According to the US EPA Method 8000, a straight-line calibration model can be used when the standard deviation of the calibration factors is less than 20%. The percent standard deviation of the calibration factors (%SD) is shown in Table 2. All BTEX compounds pass the %SD test, therefore the “single line through origin (Ax) [average calibration factor]” method is applied to the experiments conducted in this study, an example is plotted in Figure 6. The calibration equation is represented by y = mx, where y is detector response (peak area), m is the average calibration factor, and the x is concentration of analyte. We performed the calibration measurements on three different days and calculated the calibration equation (Table 2) for each day. We observed no significant differences in calibration curves amongst days. We performed a two tailed t-Test assuming unequal variances of the peak areas. The *p*-values are greater than 0.05 between dates showing that the calibration equation has a less than 5% chance of being different.

The saturation at elevated levels of BTEX puts into question whether the calibration curve will remain true in polluted atmospheres. We find that linearity holds true (%SD below 20%) for calibration curves ranging from 0–100 ppb. This informs us that we can calibrate up to 100 ppb using the straight-line calibration model and will have to consider other fitting methods for calibrations above 100 ppb, such as least squares, parabolic or quadratic, options within the PeakSimple Software.

#### 3.1.3. Detection Limit

Detection limits for each BTEX compound were determined based on analysis of 7 replicate samples of the 1 ppb BTEX gas standard (Table 3). The detection limit (DL) was defined as 3.143 times the standard deviation (SD) of the 7 replicates (DL = SD × 3.143) following the recommended protocol from CARB [42]. The sample volume at which the detection limit was computed was at 1 L, which represents the volume at which field measurements will be taken. A 1 ppb BTEX sample was introduced into the GC inlet through a Tedlar bag followed by a cleaning step. The detection limit of the GC system depends on preconcentration of the sample. A lower limit of detection can be achieved by introducing larger sampling volumes; however as seen in Figure 5, linearity may not hold beyond sample volumes of 2 L. With a 1 L sampling volume we achieve sub-ppb level detection limits on all compounds for both GC’s.

#### 3.1.4. System Drift

We evaluated the possibility of the system drift in time using a 1 ppb gas standard to assess instrument response. Table 4 shows the peak area of benzene with the standard deviation of the mean where replicates when performed. The random variation suggests there was no systematic drift over this period of time, however more experiments are required for to explore drift at a shorter time scale.

#### 3.1.5. Humidity Effects

Water vapor and condensation is known to reduce PID lamp response, therefore relative humidity (RH) effects on detection signal were explored. In the first experiment, we explore the influence that moist carrier gas may have on the analyte detection signal by removing the Nafion dryer from the system. We measured the same BTEX gas standards with moist carrier gas and dry carrier gas. We find that a moist carrier gas reduces the peak areas by 39.5%, 31.9% and 67.6% for benzene, toluene, and ethylbenzene-xylenes, respectively. This experiment demonstrates the importance of drying the carrier gas and the effectiveness of the Nafion dyer for reducing water vapor interferences.

**Figure 7:**
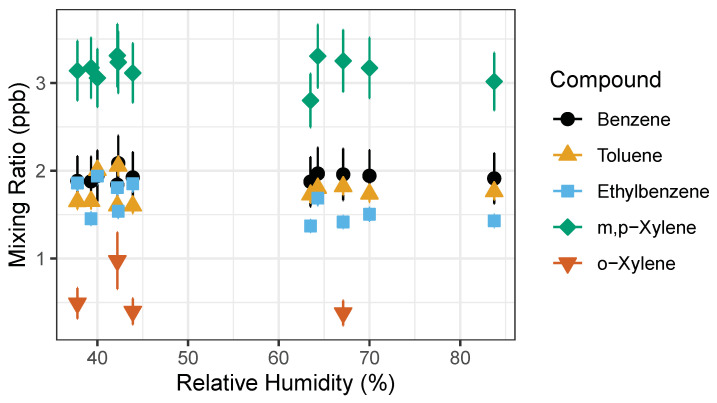
BTEX measured concentration of 1 ppb gas standard. Measured pre-rain event relative humidity (RH) was 30–50%, while the post-rain event occurred during the increased relative humidity >50% RH. Error bars represent propagation of uncertainties from gas standard and random instrument error.

The second experiment was performed outdoors during a precipitation event where humidity levels were recorded to be higher than usual (Figure 7). Ambient outdoor air was used as the carrier gas and dried as it passed through sample stream dryer (Nafion dryer in desiccant). We repeatedly measured the 1 ppb gas standard while using outdoor ambient air as a carrier gas. This experiment explored whether high humidity would affect detected signal isolating the influence of water vapor and possible condensation on the PID lamp. The measured RH ranged from 40% to 80%, pressure and temperature varied as well. A paired *t*-Test was performed on the measured concentrations pre-precipitation and post-precipitation event to test whether the rain events were significantly different. The *t*-test showed there was no statistically significant difference between pre- and post- rain event in measured benzene and toluene mixing ratios with two-tail *p*-values of 0.4255 and 0.0853, respectively. However, the t-Test showed statistically significant difference in ethylbenzene, m,p-xylenes and o-xylene measured before and after precipitation event with two-tail *p*-values of 0.0133, 1.977 × 10^−6^, and 1.641 × 10^−4^. This indicates that humidity has a significant impact on the detection of ethylbenzene, m,p-xylenes and o-xylene.

#### 3.1.6. Validation with Conventional Canister Sampling

**Figure 8:**
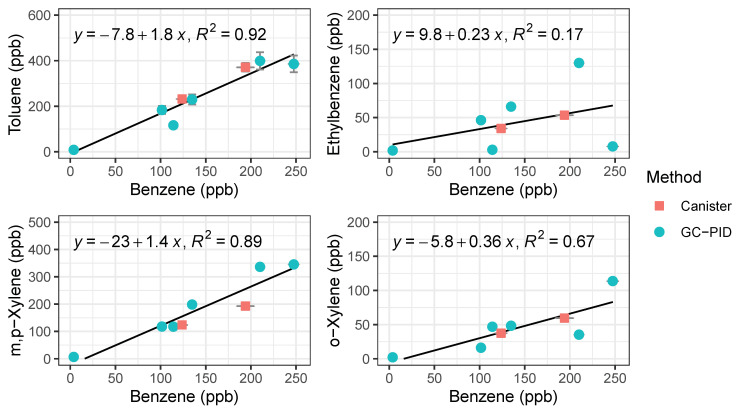
Regression plots of BTEX compounds from vehicle exhaust measured by the compact GC-PID instrument (blue) and samples collected in canisters (red) followed by analysis in a traditional benchtop GC. Error bars for the GC-PID are errors propagated from the calibration curve and error bars for the canister samples represent 5% precision accuracy from the benchtop GC.

To validate the performance of the BTEX GC-PID system, we compared the GC-PID measurements to the conventional approach of collecting air in canisters followed by measurement in the lab by a traditional benchtop GC. Two whole air samples were collected in an evacuated 2 L electropolished stainless steel canisters then returned to the University of California, Irvine for analysis of BTEX on a multicolumn, multidetector GC system further described elsewhere [43]. Vehicle exhaust from a gasoline-powered car was collected with the whole air canister at the same time that the GC’s were sampling at the same location. The canister was filled within 1 min of opening the valve while the GC trapped the sample for 2 min. While it is difficult to compare the measured BTEX values because of this difference in timing of sample collection, BTEX measurements from canisters are bracketed by the measurements made by the field-deployable GC-PID system (Figure 8). Although emissions of vehicle exhaust were not uniform in time, the slope of the non-benzene compounds to benzene in Figure 8 are similar between both measurement methods. The toluene to benzene (T/B) ratio was 1.76, within range of reported T/B literature values of close to 2 for traffic emissions in urban areas [19,32]. This gives confidence that the GC-PID instruments are not only capable of quick BTEX analyses, but also capable of measuring at high BTEX mixing ratios accurately before saturation occurs.

### 3.2. Mobile Measurements of Traffic Emissions

**Figure 9:**
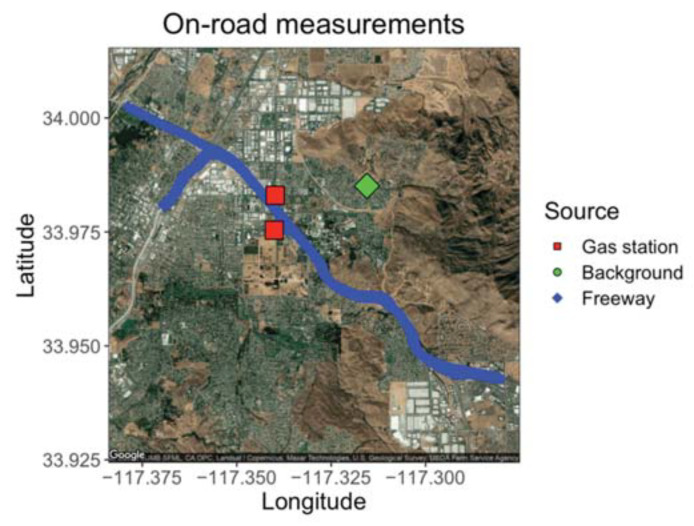
Map of freeway route on SR 60 with targeted gas stations and the local background measured 6.5 km east of the SR 60.

The GCs were placed aboard a mobile platform to demonstrate the ability of the compact GC-PID to measuring environmentally relevant BTEX patterns in a field setting. Ambient outdoor samples were drawn in from outside through Teflon tubing connected to the GC inlet. On 24 February 2020, we sampled ambient outdoor air with the GC before rush hour (15:00 to 16:00 PST) and during the afternoon rush hour (16:00 to 20:00 PST) at three locations: on the California State Route (SR) 60, a heavily trafficked multi-lane highway in Riverside, California (24 samples); a local background measurement site location 6.5 km east of the SR 60 (9 samples); and at nearby gas stations (5 samples) as shown on Figure 9. The local background site was chosen to be at a residential zone with minimum vehicle traffic and away from the major traffic source. Background measurements were taken before, during, and after the freeway transects to get an idea of the enhancement in BTEX produced by the afternoon rush hour. A GPS tracker was used alongside the GCs to measure location of measurements on and off the freeway.

**Figure 10:**
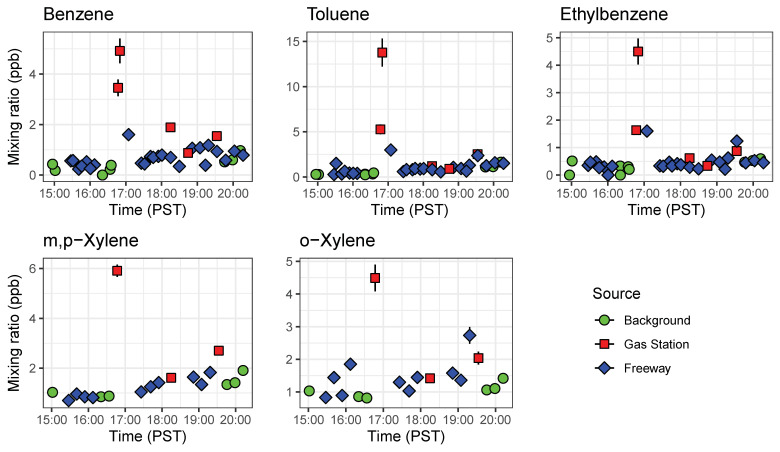
Timeseries of BTEX compounds detected by both GC1 and GC2 abroad a mobile platform before rush hour (15:00 to 16:00 PST) and during the afternoon rush hour (16:00 to 20:00 PST). These results show the portability of the compact GC-PID for mobile applications. Error bars represent error propagated from instrument calibration.

**Figure 11:**
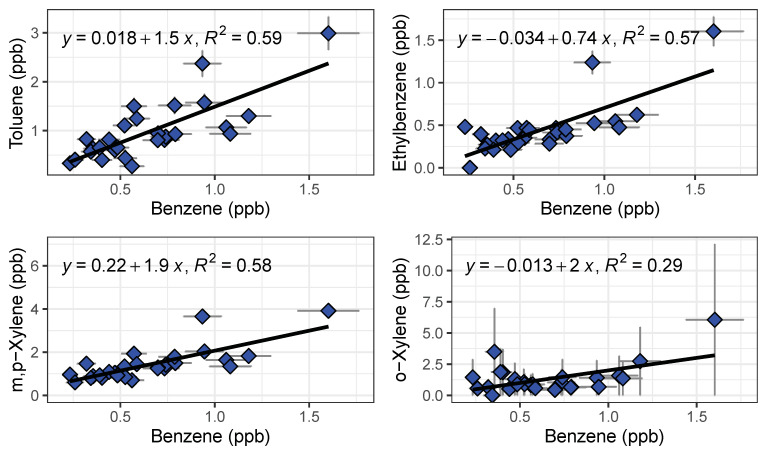
Benzene ratios for on-freeway emissions measured by GC1 and GC2 shown with a linear regression fit.

We observed systematic differences between locations and over the course of the rush hour (Figure 10). As expected, gas stations had the highest measured BTEX levels from evaporating fuels. Benzene was always higher at gas stations than on freeway or at the local background site, and other species tended to be higher at the gas station as well. On-freeway levels tended to be higher than background when comparing similar time periods, but the increase in emissions over the rush hour was larger than the differences between locations. BTEX mixing ratios increased as SR 60 became congested as the day progressed. A similar increase was observed at the background site, with close to doubling of the benzene mixing ratio from the start of rush hour to the end (Figure 10). We plotted ratios of benzene for on-freeway samples, and generally saw a strong, linear relationship between benzene and toluene, ethylbenzene and the xylene isomers, giving confidence that the emissions were emitted from the same source (Figure 11). The observed T/B ratio for on-freeway measurements was 1.47. This value is lower than what has been observed in studies from urban traffic, but in accordance with observations of a lower value when a strong diesel contribution is present [44]. The SR 60 is a main route for diesel trucks transporting goods to and from warehouses in the area.

## 4. Discussion

In this study, we demonstrated the capability of a small field-deployable GC-PID for measurements of ambient BTEX levels in field and mobile campaign settings. Although the compact build of the instrument allows for portability that is ideal for field measurements and screening analysis, there are disadvantages to having a small instrument footprint. The chassis can only accommodate a small oven, thus limiting column diameter and temperature programming. The BTEX compounds have a wide range of boiling point temperatures (80 to 138 °C), while the isothermal oven was set to 60 °C. The oven was set closer to the benzene boiling point temperature, explaining why the heavier molecules are slow to elute and have broader peaks. A longer column gives better separation; however, the size of the oven limits the length and diameter of a column. Temperature programming was not included in this design due to added cost estimated at 5k USD.

Toxic VOC’s in urban air are of low concentration in often complex mixtures. Compounds with a similar structure as benzene (cyclopentane, pentane and cyclohexane) may show up as a small peak before benzene in the chromatogram. In our experiments, small unknown peaks were observed before the benzene peak. This becomes a concern when measuring polluted atmospheres because the area under the peaks can merge, resulting in a loss of the ability to resolve benzene. In addition, when higher mixing ratios are measured, carry-over from the previous sample is observed to influence the subsequent measurement. Regular heating of the trap is recommended to remove adsorbent from the trap. Tenax-GR material is commonly used as the adsorbent material for preconcentration of BTEX, but other studies have shown that basolite C300 and ZSM-5 zeolites can be a more effective adsorbent material [39,45].

The instrument was developed for near-real time analysis, using ambient air as a carrier gas to reduce the need for consumables. The purity of the carrier gas is an important factor to consider, such as presence of VOC’s and water vapor. The Tenax-GR trap amplifies the amount of BTEX in the sample, thus when measuring in polluted atmospheres, BTEX in the carrier gas would be of a negligible amount compared to that reaching the detector desorbed off the trap. Any contamination would show up as a constant background and not as a peak. However, an addition of a carbon trap may help reduce VOC impurities in the carrier gas which can be explored in future studies [46]. Although, commonly used carrier gases like He and N_2_ are more efficient at pushing molecules through the capillary columns and give a better separation of peaks in the chromatogram, these gases need constant replacement and are not ideal to take to the field.

Humidity has been shown to decrease the detected BTEX signal [47]. We used several preventative measures in the design of the GC to remove water influence, such as: trap desorption to remove water in the sample, the precolumn backflush and the Nafion dryer which significantly decreases the amount of water in the sample. These measures all prevent water from reaching the detector; however, we saw that high humidity does indeed reduce the signal detected of ethylbenzene, m,p-xylene and o-xylene. Additionally, saturation of desiccant can affect the amount of water being removed from the stream of air. Daily calibrations done in the field include these uncertainties introduced by relative humidity and other environmental factors.

## 5. Conclusions

This study characterized the performance of a small field-deployable GC as a BTEX screening tool in the field at near-real time measurements. Working closely with the manufacturer we were able to optimize the configuration for speciation of the BTEX compounds and detect at the expected atmospheric background levels. Monitoring of BTEX background levels requires instrumentation that is sensitive to ppb or sub-ppb levels. We demonstrate the detection range of the compact GC-PID to be below 1 ppb for all BTEX compounds and up to 500 ppb. Compared to other commercial systems available and laboratory prototypes, the BTEX GC-PID performs remarkably well.

Three configurations were tested to determine the best selectivity and sensitivity. Two column configurations and flushing methods were explored: precolumn backflush method and backflush to detector. We observed a more stable baseline with the precolumn backflush; thus, we retained the precolumn to detector plumbing with capillary columns MXT-5 with 15 m length (0.53 mm ID × 0.25 µm) and MXT-1301 with 30 m length (0.53 mm ID × 0.3 µm). This strategy allows for minimal equipment and relies on ambient air as the carrier gas. We show that linear calibrations can be achieved within 0–100 ppb using the single line through origin (Ax) calibration method on PeakSimple. When expected concentrations are above this range, a non-linear method can be applied.

We demonstrate that the compact design of this GC-PID is ideal for stationary and mobile measurements. The design presents the opportunity to screen for BTEX at a higher spatial resolution with possibility of establishing dense networks of VOC measurements. Field-deployable GCs have the potential to aid in emergency air quality responses (e.g., refinery fires) and give near real-time air pollution measurements. Inexpensive GCs offer an exciting alternative to conventional bench-top equipment accessibility allowing monitoring of pollutants with higher spatial resolution in impacted communities that can aid in air quality assessments in support of current regulations (e.g., Assembly Bill 617).

## Figures and Tables

**Table 1:** BTEX GC-PID descriptions for configuration a, b, and c. Configuration a and b had the same capillary columns with different backflushing method, while configuration c retained the precolumn backflushing method with new capillary columns. Only benzene and toluene limit of detections are show for comparison purposes.

Configuration	Capillary Columns	Backflush Method	Analysis Time (min)	Limit of Detection (ppb)
a (Figure 3a)	15 m MXT-WAX 15 m MXT-1	Precolumn backflush	12	Benzene 0.09 Toluene 0.10
b (Figure 3b)	15 m MXT-WAX 15 m MXT-1	Backflush to detector	<20	Benzene 0.37 Toluene 0.11
c (Figure 3c)	15 m MXT-5 30 m MXT-1301	Precolumn backflush	<15	Benzene 0.06 Toluene 0.10

**Table 2:** Calibration curves for BTEX compounds. SD of slope indicates standard deviation of the slope in the calibration equation. A %SD lower than 20% indicates linearity exists in the calibration curve. N represents the number of points in the curve.

Compound	Date	Calibration Equation	SD of Slope	R^2^	%SD	N
Benzene	2/22/2020	*y* = 7.38*x*	1.10	0.97	7.38	4
	2/24/2020	*y* = 5.60*x*	0.80	0.97	5.67	4
	2/27/2020	*y* = 6.87*x*	1.50	0.97	6.79	3
Toluene	2/22/2020	*y* = 11.56*x*	0.60	1.00	11.11	4
	2/24/2020	*y* = 10.99*x*	0.90	1.00	11.11	4
	2/27/2020	*y* = 10.95*x*	1.30	0.97	10.74	3
Ethylbenzene	2/22/2020	*y* = 6.57*x*	0.30	0.99	6.12	4
	2/24/2020	*y* = 5.25*x*	0.80	0.97	5.00	4
	2/27/2020	*y* = 7.18*x*	1.20	0.89	7.36	3
m,p-xylene	2/22/2020	*y* = 9.24*x*	1.00	0.96	9.35	3
	2/24/2020	*y* = 8.44*x*	0.80	0.98	8.33	4
	2/27/2020	*y* = 9.13*x*	1.60	0.91	9.36	3
o-Xylene	2/22/2020	*y* = 2.80*x*	0.90	0.90	2.89	4
	2/24/2020	*y* = 2.67*x*	0.70	0.99	2.68	3
	2/27/2020	*y* = 4.23*x*	1.10	0.98	4.26	3

**Table 3:** BTEX detection limits for each BTEX GC-PID instrument referred to as GC1 and GC2.

Compound	GC1 (ppb)	GC2 (ppb)
Benzene	0.06	0.19
Toluene	0.10	0.28
Ethylbenzene	0.37	0.18
m,p-Xylene	0.33	0.32
o-Xylene	0.21	0.16

**Table 4:** Drift experiments using a 1 ppb BTEX gas standard organized by date. Observed benzene reported in area units (a.u.). For dates where number of 1 ppb gas standard samples (N) were > 1, the standard deviation is shown in parentheses.

Date	N	Benzene (a.u.)	
10/9/2019	7	13.51	(0.19)
11/25/2019	1	19.24	NA
1/7/2020	1	15.72	NA
1/14/2020	4	12.42	(0.11)
2/19/2020	1	16.92	NA
2/20/2020	3	14.94	(0.47)
2/22/2020	4	14.15	(0.51)
2/24/2020	1	10.65	NA
2/27/2020	1	13.59	NA
3/5/2020	3	11.45	(0.11)

## Data Availability

The data presented in this study are openly available in OSF at DOI, reference number 10.17605/OSF.IO/7EW8V.
